# Supplementary figures and images for: High throughput microRNAs sequencing profile of serum exosomes in women with and without polycystic ovarian syndrome
Source: PeerJ. 2021 Mar 12;9:e10998. doi: 10.7717/peerj.10998 (PMC7958896; doi:10.7717/peerj.10998)

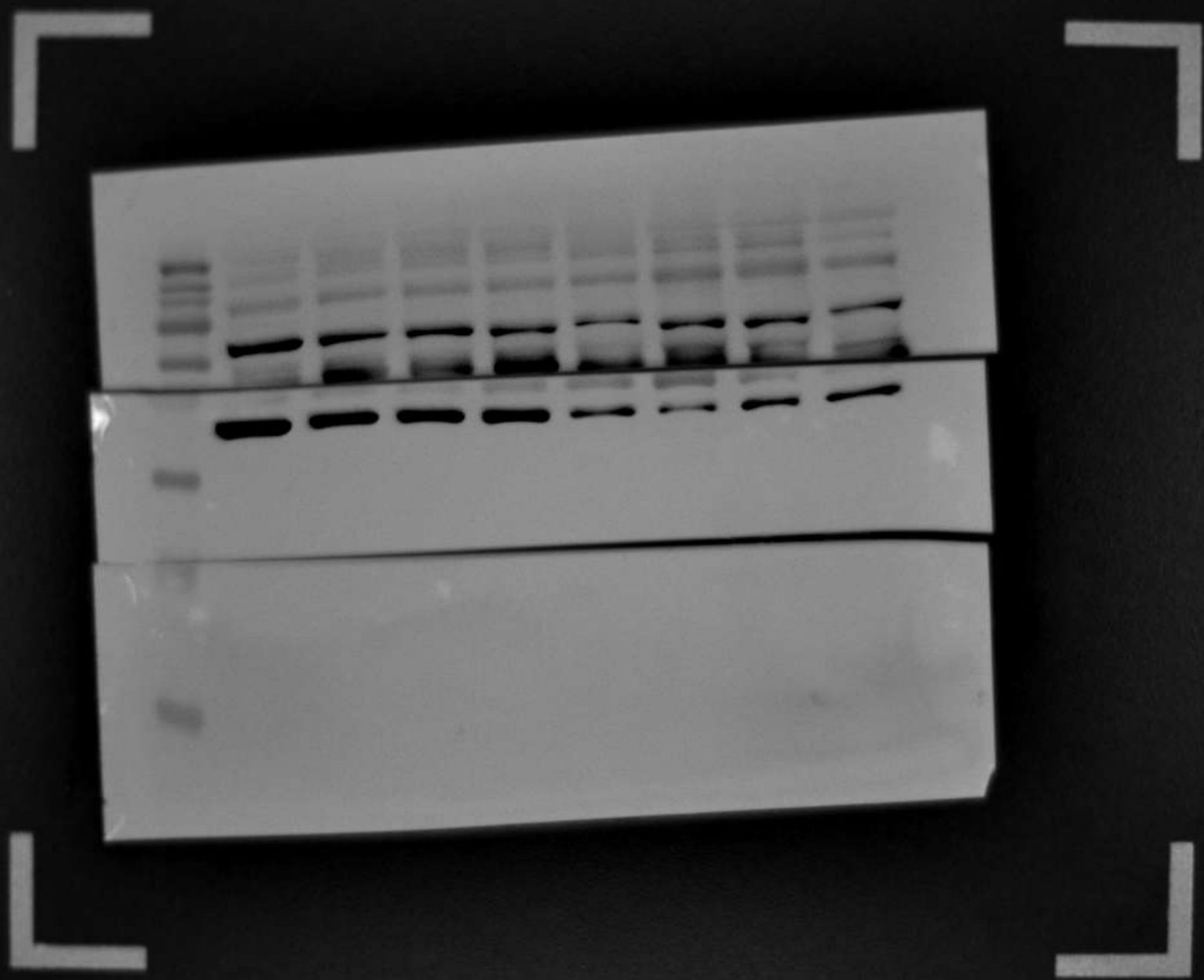

Supplement: Supplemental Information 1 [file peerj-09-10998-s001.pdf]

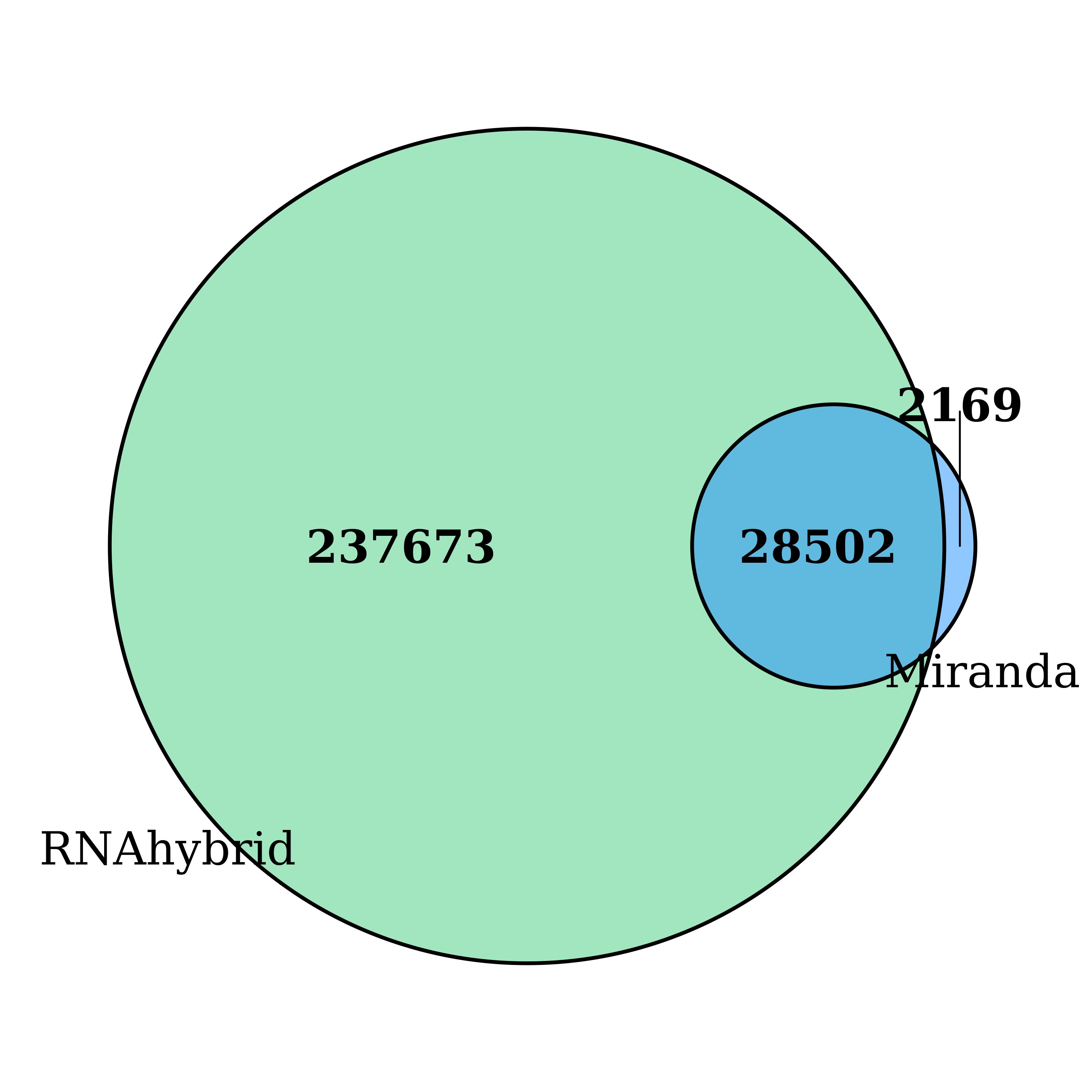

Supplement: Supplemental Information 5 [file peerj-09-10998-s005.jpg]

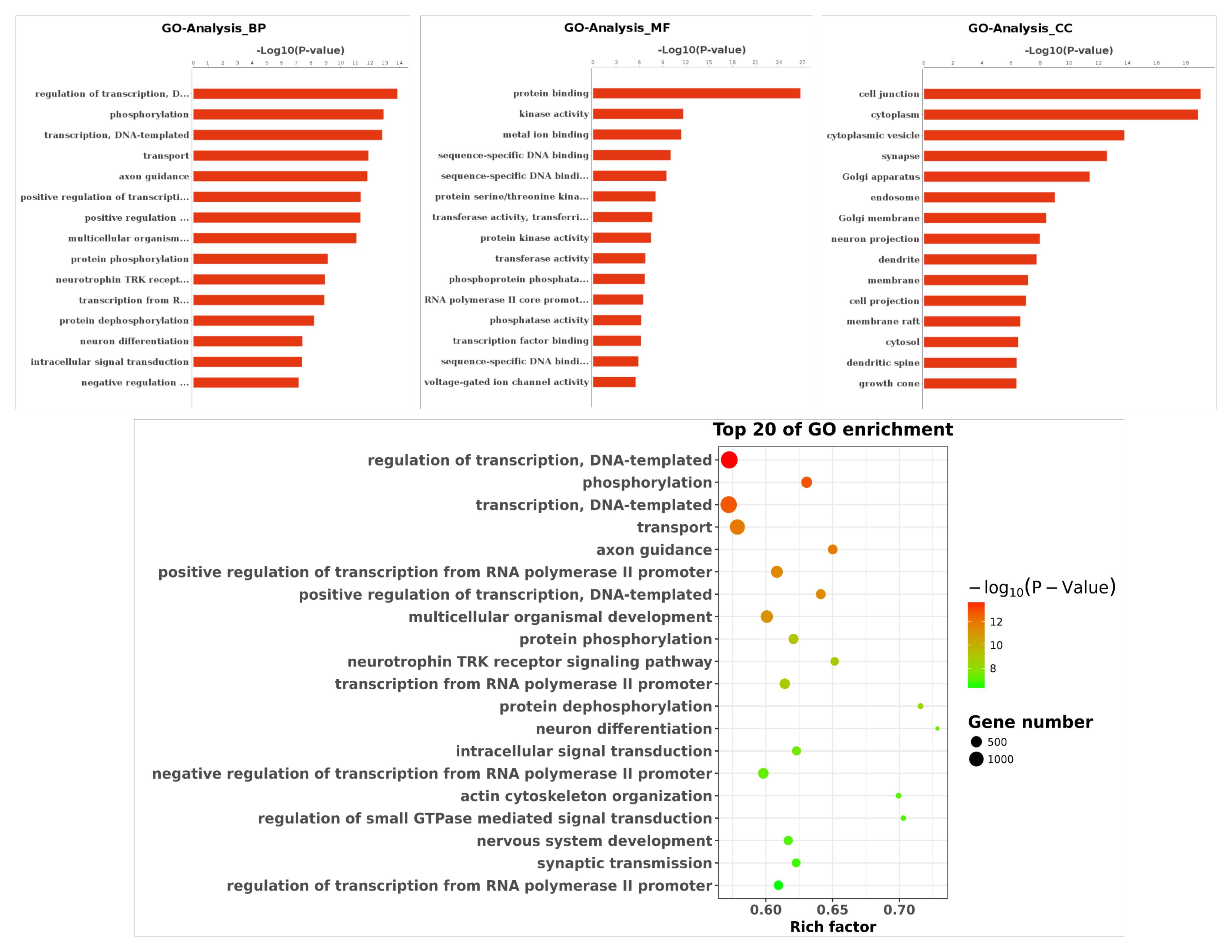

Supplement: Supplemental Information 6 — (A) GO analysis of all the differentially expressed genes included the leftmost Biological Process (BP), the intermediate Molecular Function (MF), and the rightmost Cellular Component (CC). (B) The scatter plot is a graphical display of GO enrichment analysis results. The degree of GO enrichment is measured by Rich factor, p value and the number ofgenes enriched in this pathway. Rich factor refers to the ratio of the number of differentially enriched genes to the number of annotated genes in GO. The larger the Rich factor, the greater the degree of enrichment [file peerj-09-10998-s006.jpg]

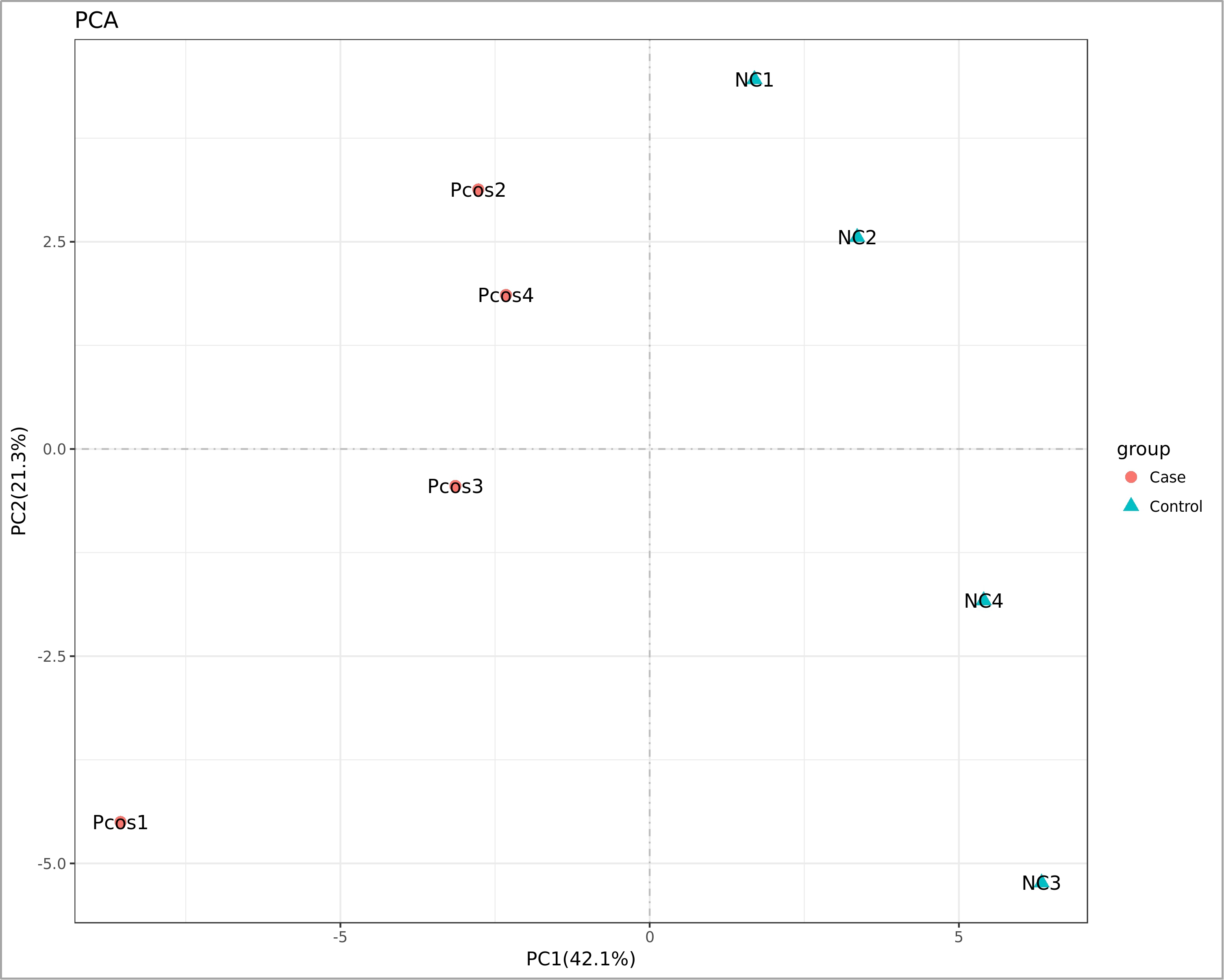

Supplement: Supplemental Information 7 [file peerj-09-10998-s007.jpg]

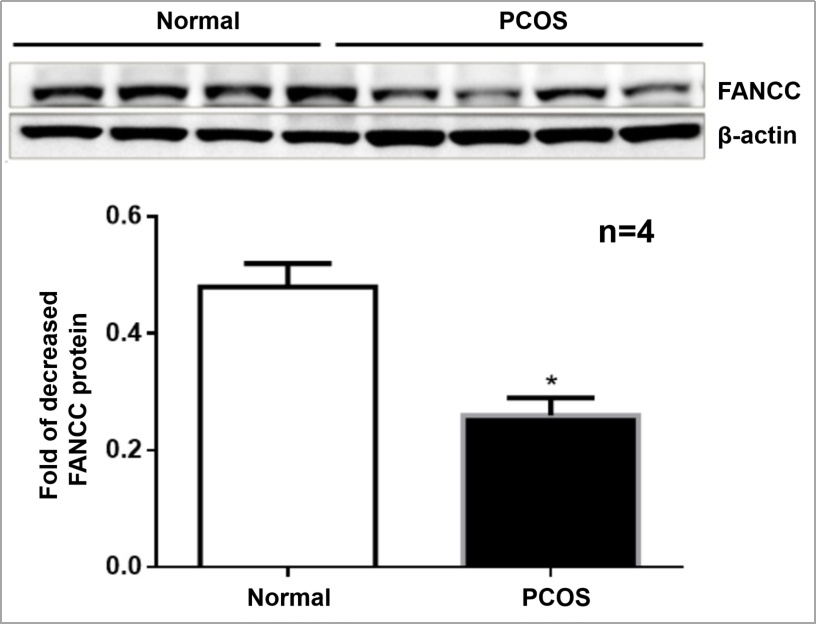

Supplement: Supplemental Information 8 [file peerj-09-10998-s008.jpg]

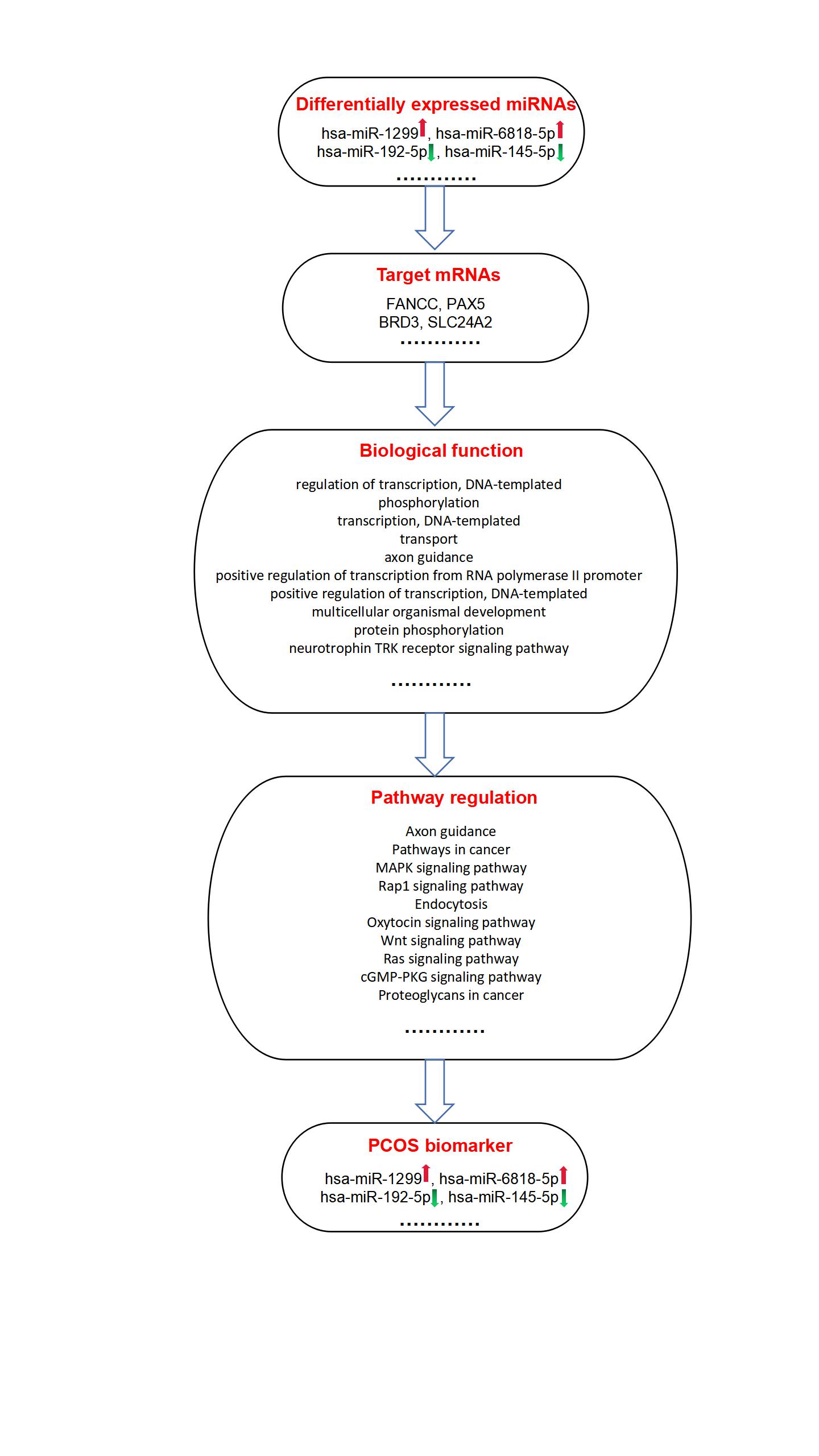

Supplement: Supplemental Information 9 [file peerj-09-10998-s009.jpg]
